# Targeting HIBCH to reprogram valine metabolism for the treatment of colorectal cancer

**DOI:** 10.1038/s41419-019-1832-6

**Published:** 2019-08-13

**Authors:** Yunlong Shan, Yuan Gao, Wei Jin, Minmin Fan, Ying Wang, Yanhong Gu, Chenxiao Shan, Lijun Sun, Xin Li, Biao Yu, Qiong Luo, Qiang Xu

**Affiliations:** 1 State Key Laboratory of Pharmaceutical Biotechnology, Nanjing Drum Tower Hospital and School of Life Sciences, Nanjing University, 210023 Nanjing, China; 2 Department of Oncology, The First Affiliated Hospital with Nanjing Medical University, 210029 Nanjing, China; 3 School of Pharmacy, Nanjing University of Chinese Medicine, 210023 Nanjing, China; 4 State Key Laboratory of Bioorganic and Natural Products Chemistry, Shanghai Institute of Organic Academy, 200032 Shanghai, China

**Keywords:** Cancer metabolism, Colorectal cancer

## Abstract

Valine catabolism is known to be essential for cancer cells but the detailed mechanism remains unclear. This study is to explore the critical roles of 3-hydroxyisobutyryl-CoA hydrolase (HIBCH) in colorectal cancers (CRC) and to develop a new therapy returning valine metabolism homeostasis. High HIBCH expression was first confirmed to correlate with poor survival in patients with CRC, which was then linked to the increased cell growth, resistant apoptosis, and decreased autophagy in CRC cells. The functions of HIBCH in CRC were dependent on its mitochondrial localization. High HIBCH level was further demonstrated to promote the metabolism of tricarboxylic acid cycle as well as oxidative phosphorylation in CRC cells. Based on above findings, we further discovered a novel valine catabolism inhibitor SBF-1. The pharmacological blockade of HIBCH mitochondrial localization with SBF-1 resulted in decreased cancer cell growth and increased autophagy, collectively contributing to the antitumor effect both in vitro and in vivo. Moreover, anti-VEGF therapy with bevacizumab increased HIBCH level in CRC cells, which in turn caused the resistance to the therapy. The interference with HIBCH function by SBF-1 significantly increased the antitumor efficacy of bevacizumab and led to a robust survival benefit. The present study identified HIBCH as a critical enzyme of valine catabolism in CRC progression and resistance to anti-VEGF therapy. We also provided a novel HIBCH inhibitor SBF-1, which highlighted the combined therapy using valine catabolic inhibitor along with anti-VEGF drugs, to control progression of CRC.

## Introduction

Colorectal cancer (CRC) is one of the leading causes of cancer-related death worldwide^1,2^, with an estimated 1.8 million cases yearly and an annual mortality of over 881,000^3^. Due to the relatively asymptomatic progression of the disease in the early stages, patients are frequently diagnosed with metastatic disease, with a 5-year survival rate of around 14%^4^. Current chemotherapies include 5-flurouracil (5-FU), oxaliplatin and irinotecan. The monoclonal antibodies including vascular endothelial growth factor (VEGF) inhibitor bevacizumab and epidermal growth factor receptor (EGFR) inhibitor cetuximab are used along with 5-FU-based therapy for metastatic CRC. Despite these therapeutic advances, the latest adjuvant clinical trials have shown that the addition of targeted agents to standard chemotherapies has no value in advanced CRC^5–8^.

The essential hallmarks of cancer are intertwined with an altered cancer cell intrinsic metabolism^9^. Metabolic changes in tumor cells directly regulate development and progression of cancer. Cancer cells adjust corresponding energy metabolism in order to fuel cell growth and division^10^. To meet the energy requirements of cancer cells, the entire metabolism, especially the tricarboxylic acid (TCA) cycle, is reorganized to augment anabolic reactions associated with cell growth and proliferation. Thus, metabolic reprogramming as a well-appreciated hallmark of cancer has been extensively used for drug discovery research^11,12^.

Branched-chain amino acids (BCAAs) including leucine, isoleucine, and valine, are essential amino acids, and they have recently emerged as predictors for the future risk of cancers and diabetes^13,14^. Two different types of tumors, specifically pancreatic ductal adenocarcinoma and non-small-cell lung carcinoma, were recently shown to exhibit different usages of BCAAs^15^. 3-hydroxyisobutyryl-CoA hydrolase (HIBCH), the enzyme which catalyzes the conversion of 3-hydroxyisobutyryl-CoA to 3-hydroxyisobutyrate^16^, is a critical mitochondrial protein in valine catabolism^17,18^. The metabolite 3-hydroxyisobutyrate is further converted to succinyl-CoA and participates in metabolism of TCA cycle (Fig. S1). Mutations in the HIBCH gene are present at low levels (0.5–6.0%) in many kinds of cancers. Moreover, it has been reported that HIBCH is increased in the patients with prostate cancer^19^. Thus, the critical roles of HIBCH in cancer cell growth and valine homeostasis suggest the potential for new therapies targeting valine metabolism by antagonizing HIBCH. In this study, we demonstrated for the first time that HIBCH played an important role in the pathogenesis of CRC. We found that a synthetic steroidal glycoside SBF-1 inhibited TCA cycle and oxidative phosphorylation (OXPHOS) by blocking mitochondrial localization of HIBCH, and thus showed its remarkable antitumor effects against CRC both in vitro and in vivo. Interestingly, SBF-1 was also able to overcome the resistance of CRC to the anti-VEGF therapy with bevacizumab by interfering mitochondrial HIBCH, which can be used as a new combined therapy for CRC.

## Results

### High HIBCH expression correlates with poor survival in patients with CRC

**Fig. 1:**
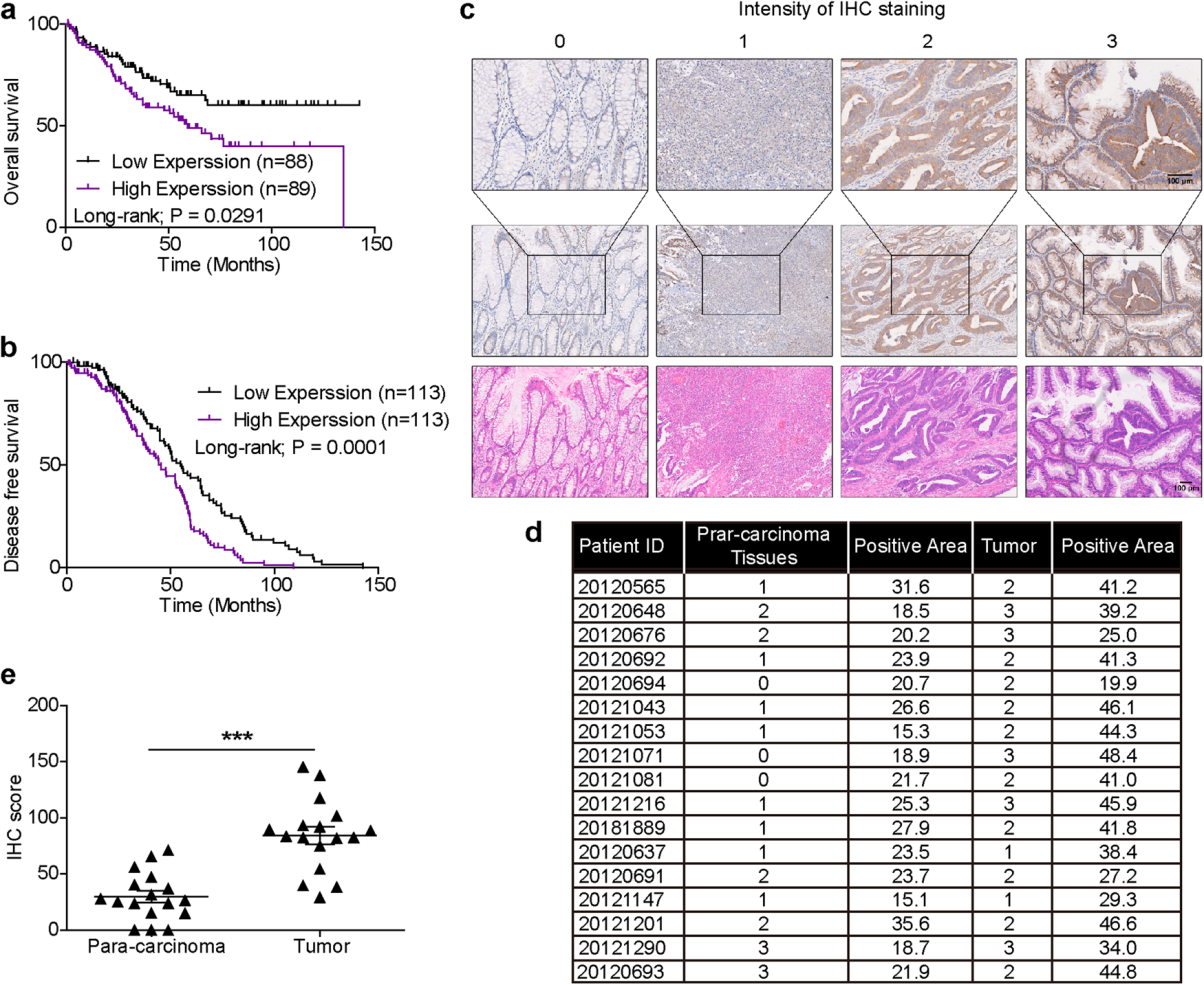
High HIBCH expression correlates with poor survival in patients with CRC. **a** HIBCH mRNA expression predicts poor survival in CRC patients in GSE17536. Patient survival data consisted of 177 CRC cases. **b** Cumulative DFS curves of HIBCH in GSE14333. Patient recurrence data consisted of 226 CRC cases. **c** Top and middle row, the protein level of HIBCH in tumors and para-carcinoma tissues from human CRCs was detected by immunohistochemistry (IHC); representative images of negative (intensity 0), weak (intensity 1), moderate (intensity 2), and strong (intensity 3) staining. Bottom row, H&E staining. Scale bars, 100 μm. **d** Summary table of IHC staining area. **e** IHC scores of HIBCH in colorectal tumor and para-carcinoma tissues were calculated as follows: IHC staining × positive area

How HIBCH influences CRC initiation and progression remains unknown. To explore the clinical relevance of HIBCH expression and CRC, we firstly analyzed the data from the Gene Expression Omnibus (GEO) datasets. Kaplan–Meier analysis showed that the increased HIBCH expression was positively related with a poor overall patient survival (Fig. 1a), and patients with higher HIBCH levels had a significantly shorter disease-free survival vs. patients with lower HIBCH levels (Fig. 1b). Then, HIBCH protein level was evaluated by immunohistochemical staining in 17 paired CRC tissues and the para-carcinoma tissues (Fig. 1c, d). We found that HIBCH expression significantly increased in the tumor tissues as compared with the para-carcinoma tissues (Fig. 1e and Fig. S2). These results suggest that high HIBCH expression may play an important role in the pathogenesis of CRC.

### HIBCH is involved in CRC cell growth and mitochondrial respiration

**Fig. 2:**
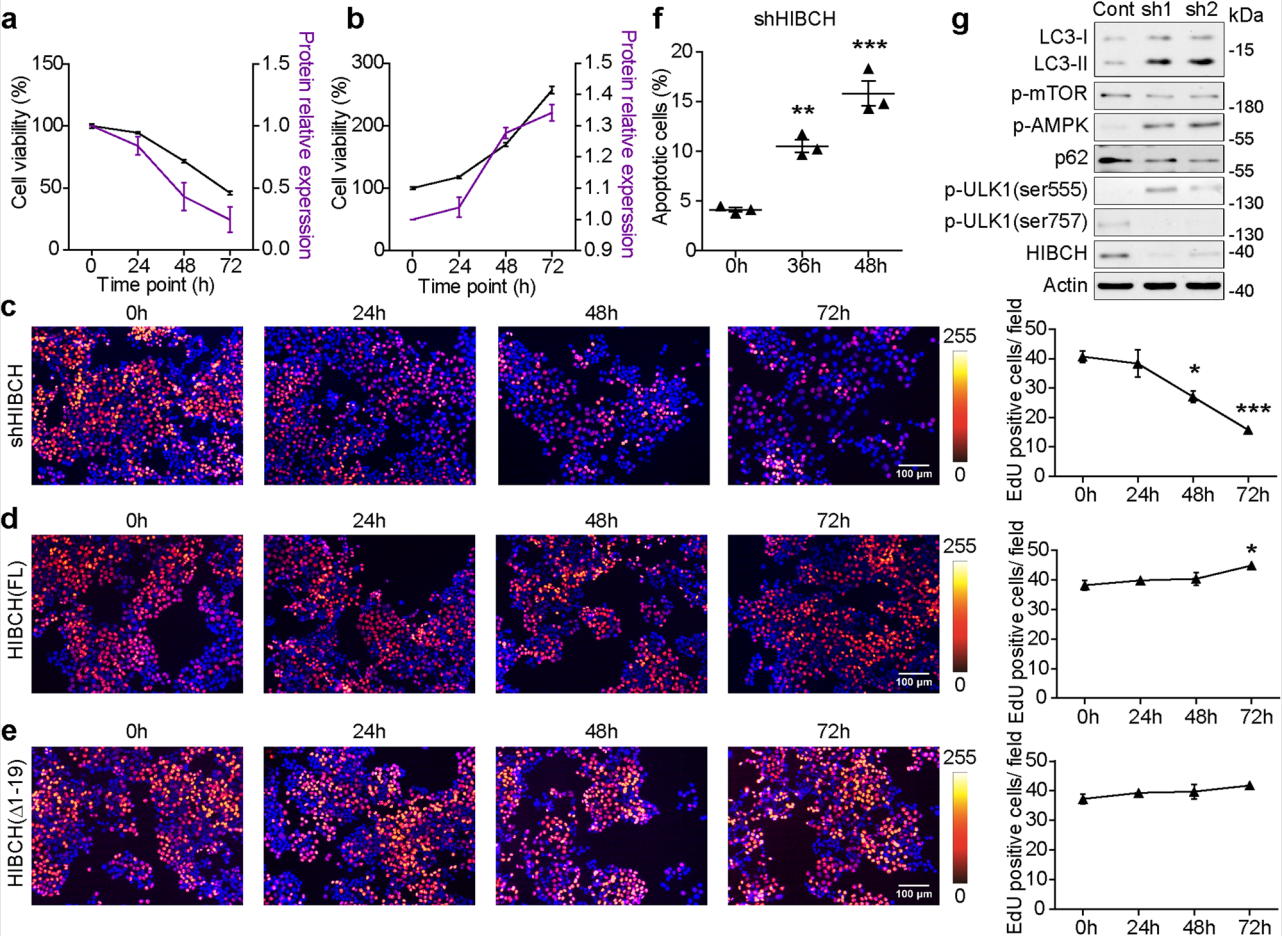
HIBCH is involved in CRC cell growth in vitro. **a**, **b** Dynamic analysis of protein expression of HIBCH and cell viability in HCT116 cells for the indicated time. A, shHIBCH; B, OE-HIBCH. **c**–**e** Left, representative images of EdU incorporation and Hoechst 33258 nuclear staining (blue) in HCT116 cells with silencing HIBCH (**c**), HIBCH (FL) full length overexpression, (**d**) and HIBCH (Δ1–19) overexpression (**e**) at indicated time points. Right, quantification of EdU incorporation. Each dot represents the average of three images. *P* values were determined by one-way ANOVA with Tukey’s correction. **f** HCT116 cells were interfered with HIBCH-shRNA for the indicated time. Then the apoptosis was determined by Annexin V/PI staining. Percentage of Annexin V positive and PI-positive cells was shown. *P* values were determined by one-way ANOVA with Tukey’s correction. **g** HCT116 cells were interfered with HIBCH-shRNA for 48 h. The protein levels of LC3, p-mTOR, p-AMPK, p62, p-ULK1, and HIBCH were determined by western blotting. Data are mean ± SEM of three independent experiments. *P < 0.05, **P < 0.01, ***P < 0.001

To investigate the function of HIBCH in CRC, we overexpressed and silenced HIBCH in HCT116 cells (a colorectal carcinoma cell line). As shown in Fig. 2a, b, silence of HIBCH inhibited the viability of HCT116 cells, whereas HIBCH overexpression promoted the cell viability. The EdU incorporation assay demonstrated that HIBCH silence resulted in an obvious decrease in counts of EdU incorporated cells (Fig. 2c). Instead, overexpression of HIBCH in HCT116 cells presented an increase in the percentage of EdU-positive cells in a time-dependent manner (Fig. 2d). HIBCH is a nuclear-encoded mitochondrial enzyme with an N-terminal transit peptide, which is required for localization to the mitochondrion (Fig. S3a). When the transit peptide was removed (ΔHIBCH), the cell proliferation promoted by HIBCH overexpression was diminished (Fig. 2e).

Flow cytometric analysis of cell apoptosis showed that HIBCH knockdown induced apoptosis of HCT116 cells (Fig. 2f and Fig. S3b). It is reported that autophagy is an alternative cell death-related pathway. Therefore, we examined the protein levels of LC3 and p62 (two widely used markers of autophagy) to determine whether HIBCH regulated autophagy of CRC. LC3 conversion (LC3-I to LC3-II) and p62 degradation were found in the HIBCH deficiency HCT116 cells, as compared with the control cells (Fig. 2g). Next, we analyzed the impact of HIBCH on AMP-activated protein kinase (AMPK), which is activated in response to nutrient- and energy-poor conditions^20^ and responsible for the initiation step of autophagy. As a result, silence of HIBCH increased AMPK phosphorylation, decreased mTOR phosphorylation, and concomitantly induced the activation of autophagy-initiating complex component ULK1^21^. In particular, we found increased ULK1 phosphorylation levels at Ser555 (active ULK1 form) and decreased levels at Ser757 (inactive ULK1 form) (Fig. 2g and Fig. S3c). Overall, mitochondrial HIBCH is required for CRC cell growth.

**Fig. 3:**
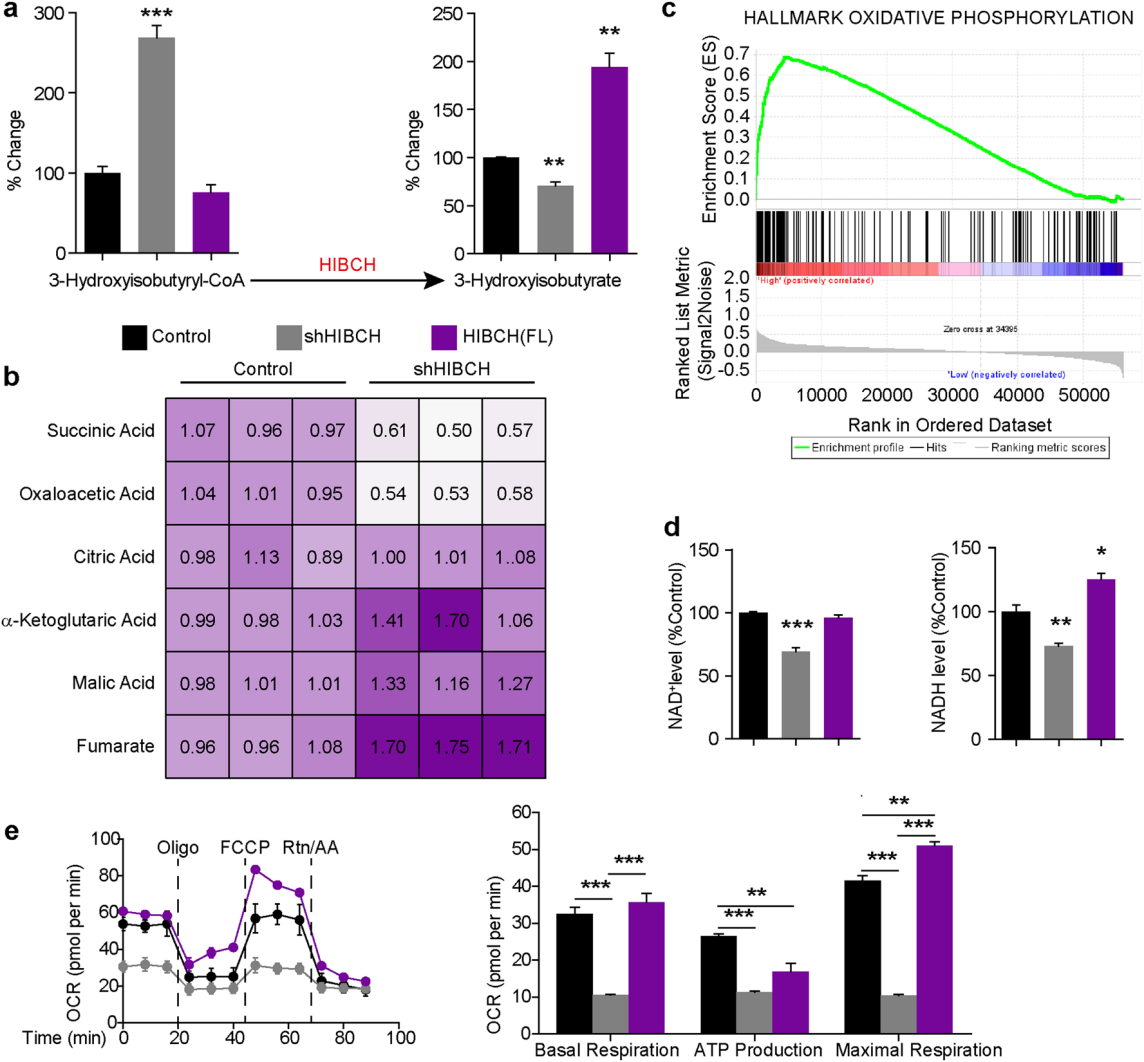
HIBCH deficiency results in decreased TCA cycle and OXPHOS in CRC cells. HCT116 cells were overexpressed with HIBCH vector or interfered with HIBCH-shRNA for 48 h. **a** 3-hydroxyisobutyryl-CoA (left) and 3-hydroxyisobutyrate (right) levels in cells were measured by UFLC–MS. *P* values were determined by Student’s *t* test. **b** Metabolites of the TCA cycle in HCT116 cells were quantified by UFLC–MS as indicated treatment. *P* values were determined by Student’s *t* test. **c** Gene set enrichment analysis using a gene set representing all genes significantly higher expressed HIBCH in CRC patients from TCGA compared with low expression, comparing cumulative expression levels from oxidative phosphorylation hallmark. **d** NAD^+^ (left) and NADH (right) levels in HCT116 cells as indicated treatment were measured by EnVision multimode plate reader. *P* values were determined by one-way ANOVA with Tukey’s correction. **e** Right, the OCR in HCT116 cells in the presence of the indicated treatment was measured by Seahorse XF Analyzers; left, graph quantification of basal respiration, ATP production, and maximal respiration from each group. Data are mean ± SEM of three independent experiments. *P < 0.05, **P < 0.01, ***P < 0.001

Autophagy is often triggered by a cellular metabolic imbalance. Therefore, we determined whether there was an altered metabolic state caused by HIBCH deficiency or overexpression in HCT116 cells. Using ultrafast liquid chromatography–mass spectrometry (UFLC–MS) analysis, we found that the generation of 3-hydroxyisobutyrate was decreased while excess 3-hydroxyisobutyryl-CoA was detected by knockdown of HIBCH, indicating that intracellular valine catabolism was altered in these cells. In contrast, HIBCH overexpression resulted in abundant 3-hydroxyisobutyrate (Fig. 3a and Fig. S4a). In valine catabolism, 3-hydroxyisobutyrate is further converted to final product propionyl-CoA, and then to succinyl-CoA via a series of enzymatic reactions and participates in metabolism of TCA cycle. We found that HIBCH knockdown resulted in decreased propionyl-CoA, while HIBCH overexpression greatly increased the levels of propionyl-CoA (Fig. S4b). We then found that HIBCH knockdown resulted in significant reduction in oxaloacetic acid and succinic acid (Fig. 3b), the metabolite of succinyl-CoA in TCA cycle. However, lack of HIBCH expression led to accumulation of fumarate and malic acid (Fig. 3b). It was reported that mitochondrial damage increased the level of fumarate during ischemia^22^. Previous studies also reported that HIBCH was important for modulating the toxic concentration of mitochondrial methacrylyl-CoA, which occurred in the middle part of valine catabolism and highly reacted with free thiol compounds, resulting in mitochondrial damage^23,24^. It is likely that lack of HIBCH will cause mitochondrial damage, leading to the accumulation of fumarate and malic acid. These results suggested that HIBCH deficiency might affect the activity of TCA cycle in CRC cells.

To further determine the potential downstream pathway of HIBCH, we performed gene set enrichment analysis (GSEA) for oncogenic signatures in Molecular Signature Database (MSigDB). GSEA of colorectal adenocarcinoma data set from TCGA showed that oxidative phosphorylation signature was enriched in the samples with high expression of HIBCH (Fig. 3c). We found that HIBCH overexpression increased intracellular NADH levels. In contrast, shRNA-mediated depletion of HIBCH resulted in significant reduction of intracellular NAD^+^ and NADH levels in cells (Fig. 3d). Next, we set out to determine the role of HIBCH in oxygen consumption. We compared the oxygen consumption rate (OCR) in the control HCT116 cells to that of transfected cells, and found that HIBCH knockdown decreased mitochondrial respiration, spare respiratory capacity, and ATP production, whereas HIBCH overexpression increased OCR (Fig. 3e), thus indicating that HIBCH increased oxidative phosphorylation activity in CRC cells.

### Decreasing HIBCH mitochondrial localization by SBF-1 contributes to the inhibition of CRC cell growth

**Fig. 4:**
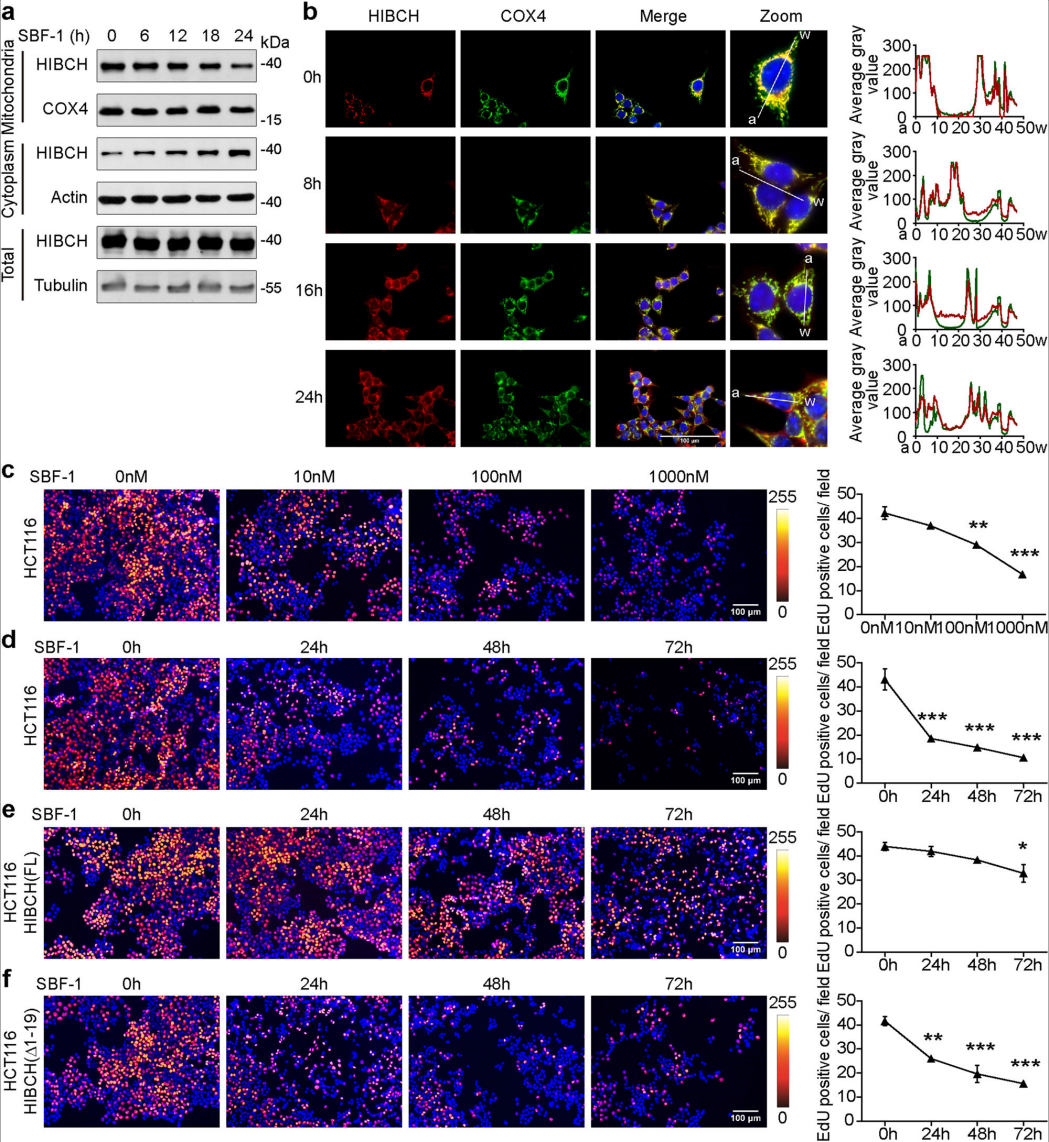
Decreasing HIBCH mitochondrial localization by SBF-1 contributes to the inhibition of CRC cell growth. **a**, **b** HCT116 cells were incubated with SBF-1 (1000 nM) for the indicated time. **a** The protein levels of HIBCH in mitochondria and cytoplasm were determined by western blotting. **b** Left, HIBCH (red), and COX4 (green) in HCT116 cells were detected by confocal immunofluorescence microscopy (blue, 4′,6-diamidino-2-phenylindole (DAPI)). The overlay is indicated in yellow. Right, line-scan analysis (by Image J RGB Profile Plot plugin) along the solid white lines depicted in the merged images to the left. **c** HCT116 cells were incubated with SBF-1 (10, 100, and 1000 nM) for 48 h. Left, representative images of EdU incorporation and Hoechst 33258 nuclear staining (blue). Right, corresponding quantification of the EdU incorporation. *P* values were determined by one-way ANOVA with Tukey’s correction. **d** HCT116 cells were incubated with SBF-1 (1000 nM) for the indicated time. Left, representative images of EdU incorporation and Hoechst 33258 nuclear staining (blue). Right, quantification of EdU incorporation. *P* values were determined by one-way ANOVA with Tukey’s correction. **e**, **f** HCT116 cells were overexpressed with HIBCH vector (**e**) or ΔHIBCH vector (**f**) for 24 h. Then cells were incubated with SBF-1 (1000 nM) for the indicated time. Left, representative images of EdU incorporation. Right, quantification of EdU incorporation. *P* values were determined by one-way ANOVA with Tukey’s correction. Data are mean ± SEM of three images. *P < 0.05, **P < 0.01, ***P < 0.001

To examine the possibility to target HIBCH for the treatment of CRC, we screened some small molecule compounds on HIBCH expression and function and found that the synthetic steroidal glycoside SBF-1 showed a unique function against HIBCH. Namely, it time-dependently inhibited the localization of HIBCH to mitochondria without affecting total HIBCH expression by both immunoblot assay and immunofluorescence staining (Fig. 4a, b). Computational modeling of SBF-1-HIBCH suggested that the side chain of SBF-1 impaired the recognition of TOM20 localized in the mitochondrial outer membrane (Fig. S5a). Then we measured binding of SBF-1 to GFP-HIBCH using microscale thermophoresis. Normalization and fitting of data from three independent measurements demonstrated that SBF-1 binds to HIBCH with a K_D_ of 35.878 μM (Fig. S5b). Considering the AKT and Ca^2+^ homeostasis inhibition of SBF-1, we investigated the effect of AKT inhibitor (MK2206) or ER Ca^2+^-ATPase inhibitor (thapsigargin, TG) on HIBCH localization. We found that MK2206 and TG failed to induce HIBCH mitochondrial translocation (Fig. S5c). SBF-1 inhibited the proliferation of HCT116 cells in a dose- and time- dependent manner (Fig. 4c, d, Fig. S5d) and induced apoptosis in HCT116 cells (Fig. S5e). To further demonstrate the role of HIBCH in the antitumor effects of SBF-1, we overexpressed HIBCH and then treated with SBF-1 in HCT116 cells. As shown in Fig. 4e, HIBCH overexpression rescued the reduction of EdU incorporation induced by SBF-1, whereas ΔHIBCH did not have this effect (Fig. 4f). The above results revealed that SBF-1 decreased CRC cell proliferation by inhibiting HIBCH mitochondrial localization.

### SBF-1 reprograms both TCA cycle metabolism and oxidative phosphorylation dependent on HIBCH in CRC cells

**Fig. 5:**
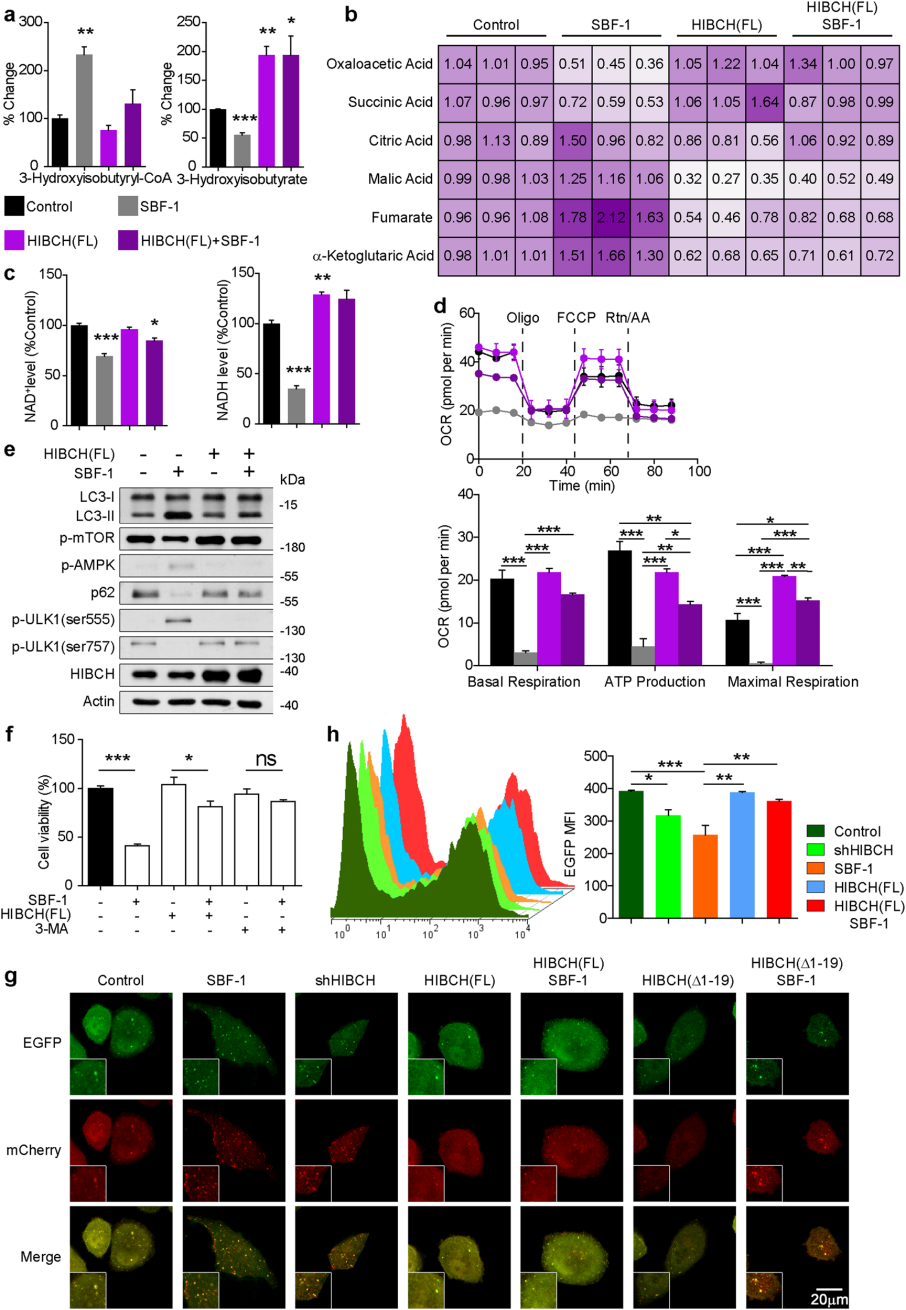
SBF-1 reprograms both TCA cycle metabolism and oxidative phosphorylation dependent on HIBCH in CRC cells. HCT116 cells were overexpressed with HIBCH vector for 24 h. Then cells were incubated with DMSO or SBF-1 (1000 nM) for 24 h. **a** 3-hydroxyisobutyryl-CoA and 3-hydroxyisobutyrate levels were measured by UFLC–MS. *P* values were determined by Student’s *t* test. **b** Metabolites of the TCA cycle were quantified by UFLC–MS. *P* values were determined by Student’s *t* test. **c** NAD^+^ and NADH levels were analysis by EnVision multimode plate reader. *P* values were determined by Student’s *t* test. **d** Top, OCR was measured by Seahorse XF Analyzers; bottom, graph quantification of basal respiration, ATP production, and maximal respiration from each group. **e** The protein levels of LC3, p-mTOR, p-AMPK, p62, p-ULK1, and HIBCH were determined by western blotting. **f** HCT116 cells were overexpressed with HIBCH vector for 24 h. Then cells were incubated with DMSO or SBF-1 (1000 nM) or plus with autophagy inhibitor 3-methyladenine (3-MA) for 72 h. Cell viability was determined by the MTT assay. Data are mean ± SEM of three independent experiments. *P* values were determined by Student’s *t* test. **g** HCT116 cells were transfected with plasmid expressing a tandem mCherry-EGFP-LC3B under different treatment conditions. Representative photomicrographs showed mCherry-LC3 and EGFP-LC3. **h** The mean fluorescence intensity analysis by flow cytometry from HCT116 in the indicated groups. *P* values were determined by Student’s *t* test. Data are mean ± SEM of three independent experiments. *P < 0.05, **P < 0.01, ***P < 0.001

To address whether the change in HIBCH localization induced by SBF-1 has any effect on cellular metabolism, we first examined the effect of SBF-1 on valine catabolism. As shown in Fig. 5a and Fig. S4c, treatment with SBF-1 (1000 nM) decreased the generation of 3-hydroxyisobutyrate and propionyl-CoA, whereas 3-hydroxyisobutyryl-CoA levels were increased. HIBCH overexpression rescued SBF-1-inhibited the hydrolysis of 3-hydroxyisobutyryl-CoA (Fig. 5a). Incubation of SBF-1 resulted in a significant decrease of succinic acid in TCA cycle, whereas overexpression of HIBCH increased the level of succinic acid in SBF-1-treated HCT116 cells (Fig. 5b). In addition, accumulation of fumarate and malic acid was also observed in SBF-1-treated HCT116 cells (Fig. 5b). Furthermore, treatment with SBF-1 significantly attenuated NAD^+^ and NADH levels (Fig. 5c) and the OCR (Fig. 5d). SBF-1-associated NADH reduction and OCR inhibition were also reversed by HIBCH overexpression (Fig. 5c, d), indicating that HIBCH was required for the suppression of oxidative phosphorylation by SBF-1 in HCT116 cells.

We then investigated the effects of SBF-1 on autophagy. As shown in Fig. 5e, LC3 conversion and p62 degradation were found in SBF-1-treated HCT116 cells, consistent with initiation of the metabolic-sensing pathway of autophagy. In addition, SBF-1 increased AMPK phosphorylation, decreased mTOR phosphorylation, and concomitantly induced the activation of ULK1. In particular, increased ULK1 phosphorylation levels at Ser555 and decreased levels at Ser757 were confirmed. The above changes in autophagic parameters induced by SBF-1 were diminished or almost completely blocked by the overexpression of HIBCH (Fig. 5e) but not ΔHIBCH (Fig. S6). Besides, the effects of SBF-1 on cell viability were rescued by autophagy inhibitor 3-methyladenine and HIBCH overexpression (Fig. 5f). We further examined the expression of a tandem mCherry-EGFP-LC3 reporter under different treatment conditions in CRC cells. Because the EGFP fluorescence signal is quenched in acidified compartments, the immature autophagosomes are visible as yellow puncta, while red-only puncta represent acidified autolysosomal structures^25^. We here found that SBF-1 treatment or HIBCH knockdown (Fig. 5g) resulted in autophagy, which was characterized by the formation of red puncta (autolysosomal) in cytoplasm. Overexpression of HIBCH reduced the formation of red puncta, but this phenomenon was not observed in cells transfected with ΔHIBCH (Fig. 5g). As expected, we validated the above results by flow cytometry (Fig. 5h). Thus, these data demonstrated the reduction of HIBCH mitochondrial localization inhibited valine catabolism, which in turn triggered autophagy in CRC cells.

### SBF-1 strongly inhibits the growth of human CRC xenografts in mice via blocking HIBCH mitochondrial localization

**Fig. 6:**
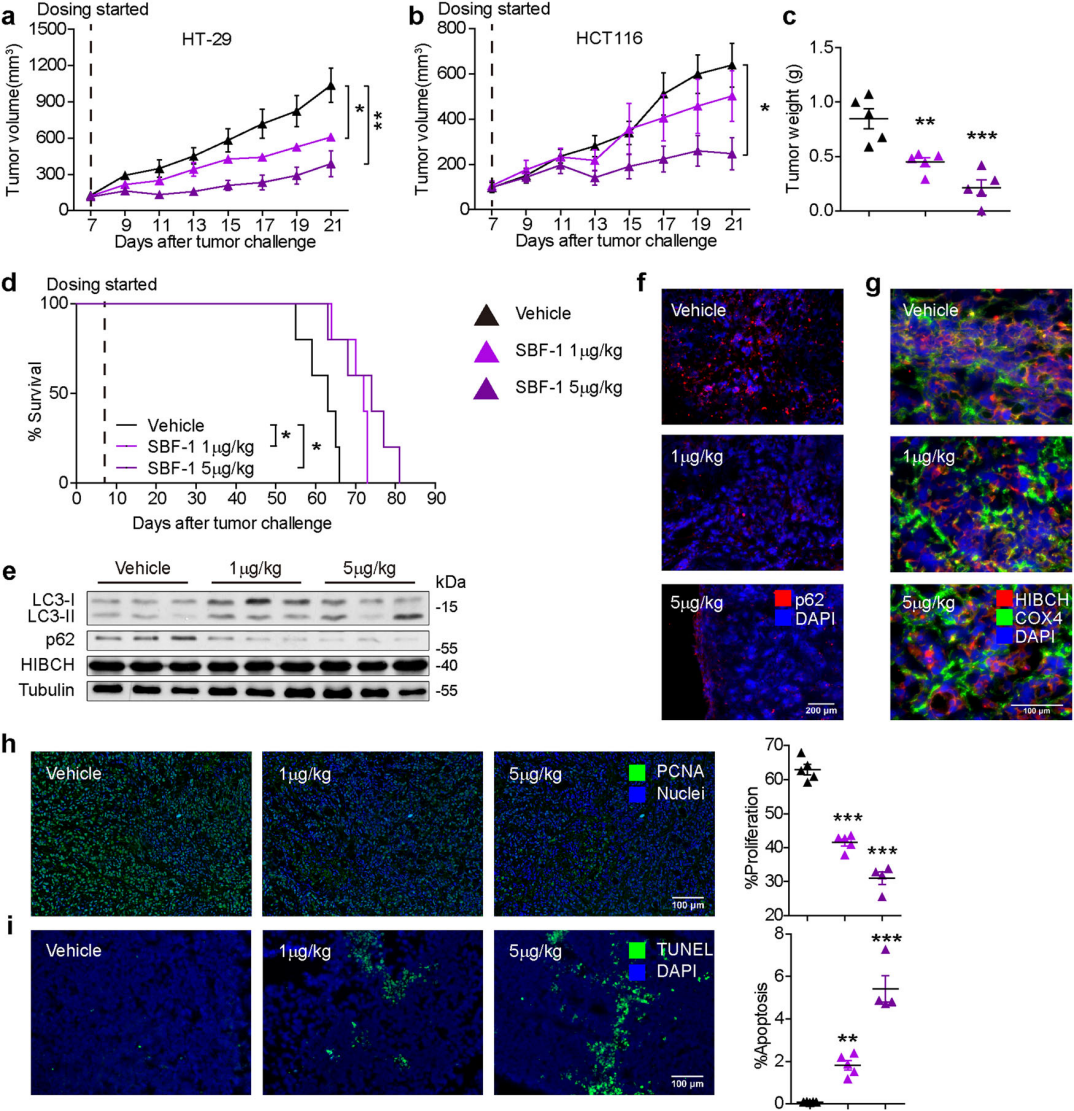
SBF-1 strongly inhibits the growth of human CRC xenografts in mice via blocking HIBCH mitochondrial localization. **a**, **b** Volumetric analysis over a 21-days treatment regimen (with vehicle or 1, 5 μg/kg SBF-1 for 2 weeks following a 5 on/2 off dosing schedule) in athymic nude mice with HT-29 **a** and HCT116 **b** cell line xenografts. *n* = 5 mice per group. *P* values were determined by Student’s *t* test. **c** Tumor weights of HT-29 xenografts at necropsy. Each dot represents one tumor. *n* = 5, *P* values were determined by Student’s *t* test. **d** Kaplan–Meier survival analysis of mice subcutaneous implanted with HCT116 cells and treated with vehicle or with 1 and 5 μg/kg SBF-1. *n* = 5. Statistical analysis is determined by log-rank test. **e** The protein levels of LC3, p62, and HIBCH in tumor samples from the indicated groups were determined by western blotting. **f** P62 in xenograft tumors from the indicated groups was detected by immunofluorescence microscopy. **g** HIBCH and COX4 in xenograft tumors in the indicated groups were detected by immunofluorescence microscopy. **h** Left, representative images of PCNA immunostaining and nuclear staining of tumors in different treatment groups. Right, relative nuclear PCNA of the indicated groups. Each symbol represents the mean of five random fields from a single mouse. Number of experimental mice: vehicle, *n* = 5; SBF-1 1 μg/kg, *n* = 5; SBF-1 5 μg/kg, *n* = 4, *P* values were determined by one-way ANOVA with Tukey’s correction. **i** Left, representative images of TUNEL and DAPI of tumors in different treatment groups. Right, relative apoptosis of the indicated groups. Each symbol represents the mean of five random fields from a single mouse. Number of experimental mice are same in **h**. *P* values were determined by one-way ANOVA with Tukey’s correction. Error bars represent ± SEM. *P < 0.05, **P < 0.01, ***P < 0.001

To evaluate the antitumor effects of SBF-1 in vivo, human CRC-bearing nude mice were intraperitoneally injected with SBF-1 for treatment. Tumor volumes and body weights were measured every 2 days. The growth of tumor xenografts and tumor weights were significantly inhibited by SBF-1 at the very low dose of 1 and 5 μg/kg in both HT-29 (Fig. 6a, c and Fig. S7a) and HCT116 (Fig. 6b) xenograft models. In mice bearing HT-29 or HCT116 CRC, only 5 μg/kg of SBF-1 slightly reduced weight gain (Fig. S7b and c). As compared with vehicle group, Kaplan–Meier analysis showed that SBF-1 significantly prolonged the overall survival of the tumor-bearing mice inoculated with HT-29 cells (median (50%) survival: vehicle, 63 days; SBF-1 1 μg/kg, 72 days; SBF-1 5 μg/kg, 74 days) (Fig. 6d). In this case, LC3 conversion (LC3-I to LC3-II) and p62 degradation in tumors were observed due to the SBF-1 treatment (Fig. 6e). Similarly, immunofluorescence staining of p62 showed a significant decrease in p62-positive cells in SBF-1-treated tumors compared with the vehicle group (Fig. 6f). Furthermore, SBF-1 treatment reduced HIBCH expression in mitochondria in tumor tissues compared with vehicle-treated xenografts (Fig. 6g). SBF-1 also significantly inhibited the expression of proliferating cell nuclear antigen (PCNA) (Fig. 6h). In contrast, terminal deoxynucleotidyl transferase dUTP nick-end labeling positive cells were significantly increased in tumors of SBF-1 treated mice (Fig. 6i). Taken together, the in vivo study strongly suggested that blocking mitochondrial localization of HIBCH by SBF-1 could be effective in inhibiting tumor growth in CRC xenograft models.

### HIBCH links between valine catabolism and anti-VEGF therapy and targeting HIBCH overcomes the bevacizumab resistance

**Fig. 7:**
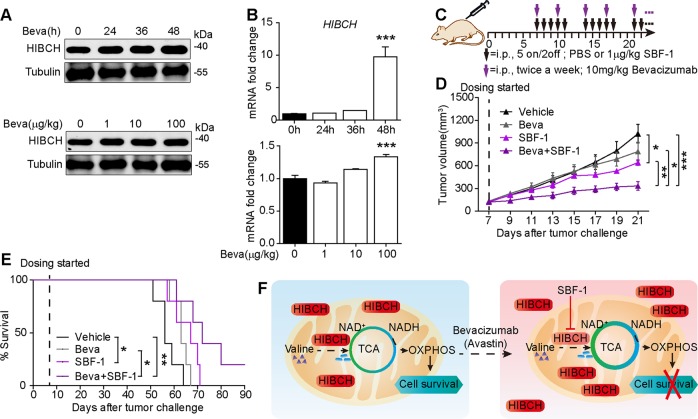
SBF-1 overcomes the bevacizumab-induced high HIBCH for the combined therapy against CRC in preclinical models. **a**, **b** HT-29 cells were treated with bevacizumab in time- (top, 100 μg/ml for the indicated time) or dose-dependent manner (bottom, 1–100 μg/ml, 48 h). The protein levels of HIBCH were determined by western blotting (**a**) and mRNA levels of HIBCH were determined by real-time qPCR (**b**). Data are mean ± SEM of three independent experiments. *P* values were determined by one-way ANOVA with Tukey’s correction. **c** Schematic of workflow for treatment as indicated. **d** Tumor volume of subcutaneous HT-29 xenograft tumors in mice from the indicated groups. *n* = 5, *P* values were determined by Student’s *t* test. **e** Kaplan–Meier survival analysis of mice subcutaneous implanted with HT-29 cells in the indicated groups. Statistical analysis by log-rank test. **f** Model showing valine catabolism utilization by the TCA cycle and OXPHOS for cells survival, which are suppressed by SBF-1 via inhibition of HIBCH mitochondrial localization. Error bars represent ± SEM. *P < 0.05, **P < 0.01, ***P < 0.001

Notwithstanding the clinical use of bevacizumab with chemotherapy for CRCs, many patients are refractory to bevacizumab^26^, and biomarkers to identify responders are missing^27^. In a recent trial, bevacizumab prolonged disease-free progression but not the overall survival^28^, and failed to show benefit in the adjuvant setting^29^. Previous studies showed that treatment with bevacizumab altered cellular metabolism in CRC cells. The upregulation of HIBCH in HT-29 tumor xenograft treated with bevacizumab was observed by global proteomic profiling^30^. This finding hints a possibility that the upregulated HIBCH may restrict the clinical efficacy of bevacizumab. Indeed, the present study demonstrated that treatment with bevacizumab elevated both protein and mRNA levels of HIBCH in CRC cells in time- and dose-dependent manners (Fig. 7a, b), which may be back to bite the effect of the drug. As expected, high HIBCH expression greatly induced bevacizumab resistance in HCT116 cells (Fig. S2d). To overcome this issue in bevacizumab, we assessed the in vivo therapeutic potential of SBF-1 and bevacizumab combination and established a protocol in the HT-29 tumor xenograft model for the combined treatment (Fig. 7c). As the result, SBF-1 as low as 1 μg/kg significantly increased the antitumor effects of bevacizumab (Fig. 7d) and prolonged the overall survival of tumor-bearing mice (Fig. 7e) without affecting the body weights of mice. Together, our findings provided potential benefits for the application of bevacizumab in CRC via combination with SBF-1.

## Discussion

The diversity of metabolic pathways that cancer cells rely on presents a key opportunity for drug development and precision medicine^31^. However, such diversity has also limited impact on the clinical management of cancer. This situation may partially arise from the fact that energy production in cancer cells is uniquely dependent on specific nutrients, such as BCAAs^13^.

The present study first found that upregulation of HIBCH occurred in CRC patients, which was associated with poor prognosis. Then we found that inhibition of HIBCH mitochondrial localization significantly reduced succinic acid in TCA cycle, decreased NADH and OCR, and concomitantly induced apoptosis and autophagy in CRC cells. Based on the evidence available, although the effect of lessened valine catabolism on NAD^+^ and NADH consuming pathways warrants further investigation, we believed that HIBCH played an important role in mitochondrial TCA cycle and OXPHOS. This could be attributed to an accumulation of toxic valine metabolites. Besides, the current study discovered that knockdown of HIBCH facilitated initiation of autophagy and inhibited proliferation in CRC cells. By transfecting with HIBCH-shRNA, LC3 conversion (LC3-I to LC3-II) and p62 degradation were observed in HCT116 cells. HIBCH deficiency resulted in a metabolic crisis, during which the energy sensor kinase AMPK was activated and then responsible for autophagy initiation. However, more in-depth investigations are required to elucidate the role of valine metabolism in CRC progression.

Another intriguing finding of this study was that a synthetic steroidal glycoside SBF-1 effectively inhibited HIBCH mitochondrial localization in CRC cells. We here identified SBF-1 as a first potent small molecule antagonist of valine catabolism. The metabolites from valine, which turn into TCA cycle, NADH levels, and OCR were all decreased by SBF-1. Elevated autophagy was another notable characteristic of the SBF-1-mediated response, which was consistent with diminished amino acid transport and metabolism^32^. SBF-1 increased AMPK phosphorylation, decreased the mTOR phosphorylation, and induced LC3-II activation and p62 degradation. Combination of SBF-1 with an autophagy inhibitor further rescued the viability of cancer cells, as elevated autophagy was likely a major response to SBF-1, potentially illuminating a future therapeutic strategy. The SBF-1-associated OCR inhibition and autophagy induction were reversed by HIBCH overexpression, but not ΔHIBCH, further indicating that mitochondrial localization of HIBCH was required for the suppression of cell growth by SBF-1 in HCT116 cells. Combined with these data, we supposed that SBF-1 might disrupt HIBCH mitochondrial localization and caused an apparent loss of HIBCH function in HCT116 cells, resulting in growth inhibition, which might explain the strong antitumor effect of SBF-1. Furthermore, in vivo studies revealed that both 1 and 5 μg/kg of SBF-1 significantly inhibited tumor growth and prolonged the overall survival of the tumor-bearing mice. Treatment with SBF-1 could promote autophagy through deceasing mitochondrial HIBCH localization. Importantly, the quantity and activity of HIBCH may represent a translational biomarker reflective of tumors likely to respond to SBF-1 and similar agents. Our study may also open opportunities for valine catabolism inhibitors for CRC therapy. Previous studies reported that HIBCH was critical for normal human development. Its mutations or deficiency inborn might lead to accumulation of toxic valine metabolites in mitochondria and cause Leigh syndrome or Leigh-like disease with mitochondrial disorders^23,24,33,34^. Given that HIBCH is overexpressed in CRC tissues, CRC cells may be more sensitive to HIBCH-targeted therapy than normal cells. And low valine diet in CRC patients will help alleviate the side effects of methacrylyl-CoA on mitochondrial damage.

Antiangiogenesis with chemotherapy did not confer a survival benefit in disease confined to lymph nodes (stage III), and the optimal use of this modality remains to be defined^35^. It is critical to unravel the mechanisms that underlie the resistance of colon cancer cells to antiangiogenic therapy. Targeting dysregulated signal transduction can be efficacious anticancer therapies with minimal adverse effects^36^. It has been demonstrated that anti-VEGF therapy with bevacizumab alters proteins involved in cellular metabolism on cancer cells, including HIBCH. Similar to David W. Greening’s results^27^, our findings pointed out the possible implications of HIBCH upregulation by bevacizumab in CRC, although further evidence is needed. Thus, interference with HIBCH function may improve the efficacy of VEGF inhibition and lead to durable antitumor responses. Strikingly, we found that combination therapy with SBF-1 and bevacizumab significantly inhibited tumor growth in CRC models, leading to a robust survival benefit. Accordingly, colorectal cancer patients treated with bevacizumab could receive combinational use of valine metabolic inhibitors. Exploration of the therapeutic opportunities of bevacizumab and SBF-1 in CRC could be promising.

BCAAs, essential amino acids promoted protein synthesis and utilization in cancer patients^37^. Leucine and isoleucine metabolism to be an important “module” within cancer metabolism, which lead to glutamate formation for biosynthesis in cancer progression^15,38,39^. Previous studies have demonstrated total BCAAs were elevated in plasma of patients with pancreatic cancers, which can be converted to acetyl-CoA and enter the TCA cycle^40,41^. However, the role of BCAAs metabolic inhibition in the cancer patients remains to be clearly defined. Branched-chain aminotransferase 1 (BCAT1), which converts BCAAs to the corresponding branched-chain α-keto acids^42,43^, has been implicated in cancer growth^44-47^. Previously, Hattori et al. reported that altered BCAAs metabolism drove cancer progression in myeloid leukemia^13^. BCAT1 enhances autophagy to induce chemoresistance^48^ in hepatocellular carcinoma cells and promote mTOR activation in breast cancer^47^. In contrast, we found that silence of HIBCH increased AMPK phosphorylation, decreased mTOR phosphorylation, and concomitantly induced autophagy in CRC. Although BCAT1 is required for tumor growth and progression in a wide range of malignancies, BCAT1 inhibition impairs all three BCAAs metabolism. Given that HIBCH is only involved in valine catabolism and bevacizumab treatment increases HIBCH protein levels in CRC, targeting HIBCH may be more specific and effective.

In conclusion, as illustrated in Fig. 7f, we demonstrated a key role of HIBCH in linking valine catabolism, TCA cycle and OXPHOS with CRC growth and found a first antagonist of HIBCH SBF-1. When CRC cells were exposed to SBF-1, the mitochondrial localization of HIBCH was suppressed, which resulted in a disturbance of valine catabolism, TCA cycle, and mitochondrial OXPHOS, activated autophagy and caused cell death. This study suggested that HIBCH could be a novel therapeutic target for CRC. Furthermore, since high HIBCH level was revealed as a self-induced reason of CRC to resist to anti-VEGF therapy, our findings also highlight a possibility of combination therapy using valine catabolic inhibitor along with anti-VEGF drugs, to control progression of advanced CRC.

## Materials and methods

### Reagents

SBF-1 was synthesized by B. Y., a coauthor, as previously reported^49^. For in vitro experiments, SBF-1 was dissolved in DMSO to a concentration of 20 mM (stock solution); and for the in vivo assay, SBF-1 was dissolved in DMSO to a concentration of 1 mg/ml (stock solution). Bevacizumab (Avastin, Roche Diagnostics GmbH, Reinach, Switzerland) was diluted in phosphate buffer solution before use, and injected i.p. at the dose of 10 mg/kg twice a week.

### Immunohistochemical staining

For IHC analysis, samples were collected and paraformaldehyde fixed, paraffin-embedded sections of tumor tissues (5 μm thick) were mounted on slides coated with 2-aminopropyltriethoxysilane, which then were baked, deparaffinized, rinsed with 3% hydrogen peroxide. After that, these sections were washed and then blocked with 3% goat serum for 5 min and subsequently incubated with an anti-HIBCH (Abcam, ab153826, Cambridge, UK; diluted 1:500) or PCNA (Santa Cruz Biotechnology, Santa Cruz, CA, USA, sc-56; diluted 1:100) antibody at 4 °C overnight. Finally, the following steps were in accordance to the protocols provided by the manufacturer of GTVisionTMIII Complex (Gene Tech, GK500705, Shanghai, China). The nucleus was stained with hematoxylin (Solarbio, G1080, Beijing, China). Sections were further mounted with neutral gums. IHC sections were photographed by Mantra 1.01 (Perkin Elmer, Waltham, MA, USA).

### Cell lines and plasmid transfection

Human colorectal cancer cells HCT116 and HT-29 were obtained from the Shanghai Institute of Cell Biology (Shanghai, China) and were maintained in DMEM supplemented with 10% FBS plus 2 mM glutamine, 100 U/ml penicillin, and 100 mg/ml streptomycin. Transient transfection of HCT116 cells were transfected with 2 μg of HIBCH plasmid or shHIBCH (sh1: CCATACAGAGTCTAAGATT; sh2: GGTTACTTCCTTGCATTAA) using the Lipofectamine® 3000 (Invitrogen, Carlsbad, CA, USA) reagents by following the manufacturer’s instructions.

### EdU incorporation assay

HCT116 were grown in 96-well plates and transfected with scramble or shHIBCH. Cells were collected at indicated time and stained with kFluor488 EdU Kit (KeyGEN Biotech, KGA331-500, Jiangsu, China) according to the manufacturer’s instructions.

### Metabolite profiling of cultured cells

Two million cells were washed three times with cold PBS and pelleted by centrifugation. Cell pellets were then flash frozen in liquid nitrogen and stored at −80 °C until further processing. Metabolites were extracted by adding 1 ml of buffer (75% of 9:1 methanol chloroform in HPLC-grade H_2_O) to the cell pellets. The lysates were incubated at 4 °C for 20 min with shaking and cleared by centrifugation at 16,800 *g* for 5 min at 4 °C. Two aliquots of supernatant were transferred to new Eppendorf tubes containing an acetonitrile/methanol (75/25, v/v) mixture. The samples were then dried-down using a SpeedVac and reconstituted with 100 μl of 50% acetonitrile in water for the analysis.

Profiling of polar metabolites was performed by UFLC–MS in positive ion mode. Profiling of TCA cycle intermediates was performed by UFLC–MS in negative ion mode as previously described^50,51^. In brief, 10 μl of reconstituted sample was loaded onto either an Xbrige C18 column (100 × 4.6 mm, 3.5 μm; Waters, Milford, MA, USA) for analysis. The metabolites were separated using a UFLC 20ADXR LC system in-line (Shimadzu Corporation, Kyoto, Japan) coupled with hybrid quadrupole time-of-flight tandem mass spectrometer (TripleTOFTM 5600 MS system, AB SCIEX Corporation, Toronto, Canada). MultiQuant software v2.1 (ABSCIEX) was used for automated peak integration and metabolite peaks were also assessed manually for quality of peak integration. UFLC–MS peak integration data of each sample were normalized to DNA content determined from a duplicate cell pellet.

### Oxygen consumption assay

Adherent HCT116 cells were seeded at 5 × 10^3^ cells/well in 200 μl of their culture medium and incubated for 24 h at 37 °C in humidified atmosphere with 5% CO_2_. After 1 h of incubation in a non-CO_2_ incubator, OCR was measured using the Seahorse Bioscience XF96 Extracellular Flux Analyzer (Seahorse Bioscience, CA, USA) where cells were subjected in sequence to the following additions: (1) basal levels were measured with no additives; (2) 1 μM oligo, which reversibly inhibits ATP synthase and OXPHOS, was added to show ATP production; (3) 0.3 μM FCCP, a mitochondrial uncoupler, was added to induce maximal respiration; (4) 0.1 μM rotenone/antimycin A was added to end the reaction. For the kinetics experiments, measurements were collected every 8 min. Basal respiration was calculated as follows: last rate measurement before first injection−nonmitochondrial respiration rate. ATP production was calculated as follows: last rate measurement before oligomycin injection−minimum rate measurement after oligomycin injection. Maximal respiration was calculated as follows: first rate measurement after FCCP injection−nonmitochondrial respiration rate. The last rate measurement after rotenone/antimycin injection standed for nonmitochondrial respiration rate^52,53^.

### Immunofluorescence (IF) staining

The samples were permeabilized in 0.1%Triton X-100 and incubated with 1% BSA/PBS to block nonspecific binding. Subsequently, the cells were immunostained by incubating with HIBCH antibody (Abcam, ab153826; diluted 1:100), COX4 antibody (Santa Cruz Biotechnology, sc-376731; diluted 1:100), and p62 (diluted 1:100) overnight at 4 °C. After being washed with PBS, cells were incubated with goat anti-rabbit AF594 (Thermo Fisher Scientific, Waltham, MA, USA, A-11037; diluted 1:500); donkey anti-mouse AF488 (Thermo Fisher Scientific, R37114; diluted 1:500). And nuclei were counterstained with DAPI (Beyotime, Shanghai, China; C1006). Fluorescent images were taken and analyzed using the ZEN pro 2012 imaging software on a Zeiss invert microscope under 100–630-fold magnification.

### Image analysis

Line-scan analysis was performed using “RGB Profiler Plot” plugin as described previously^54^. Taken briefly, raw confocal images were background corrected. A straight line with “2” width was drawn in regions of interest. Then the lines were directly analyzed by choosing Image J plugin Graphics’s “RGB Profiler Plot” under the “Plugins” menu. In the output of “RGB Profiler”, data were exported into Microsoft Excel. The length of line was plotted on the *x*-axis, while the intensity was plotted on the *y*-axis.

### Mouse experiment

Female BALB/c nude mice (6–8 weeks old, 18–20 g) were purchased from the Model Animal Genetics Research Center of Nanjing University (Nanjing, China). HCT116 and HT-29 tumors were generated by injection of 1 × 10^7^ cancer cells subcutaneously into the right flank of nude mice. The tumors were grown for several weeks, either according to a “survival” schedule (endpoint defined by tumor volume) or a fixed timepoint (endpoint defined as fixed time lapsed after tumor challenge). Tumor size was determined by caliper measurements and calculated using the formula: *V* = 0.5 × [*L* × *S*^2^], where *S* is the shorter and *L* is the longer tumor axis. Tumor weight measurements were performed postmortem. All efforts were made to minimize the animals’ suffering and to reduce the number of animals used. The medical ethical committee of Nanjing University approved all described studies.

### Preclinical mouse trials and drug regimens

Vehicle (0.1% DMSO in PBS, *n* = 5), 1 and 5 μg/kg SBF-1 (*n* = 5) were intraperitoneally injected to the tumor-bearing mice for 14 days. Vehicle and control IgG were administered in 5 days on and 2 days off (5 on/2 off, QD × 5). Treatment of nude mice carrying subcutaneous tumors was started when the tumors became palpable and had a mean volume of 50–100 mm^3^.

### Histological assessment

The samples were isolated and part of the fresh tissues were fixed in 4% paraformaldehyde and sent for hematoxylin and eosin (H&E) staining. Sections were photographed by Mantra 1.01 (Perkin Elmer) under 100-fold magnification.

### Statistical analysis

Analysis of experiments with more than two groups was performed using one-way ANOVA with Tukey’s correction for multiple comparisons, unless indicated otherwise. For experiments with two groups, statistical analysis was performed using Student’s *t* test with 95% confidence interval. Statistical analyses in the survival experiments were performed by log-rank (MantelCox) test. Statistical significance is indicated in the figures as follows: *0.01 ≤ *P* < 0.05; **0.001 ≤ *P* < 0.01; ***P < 0.001, *P* values > 0.05 are not indicated.

Other methods: Please see Supplementary Materials and Methods.

## Supplementary information

Supplemental materials and figures
