# Supplementary material for: Targeting HIBCH to reprogram valine metabolism for the treatment of colorectal cancer
Source: Cell Death Dis. 2019 Aug 13;10(8):618. doi: 10.1038/s41419-019-1832-6 (PMC6692300; doi:10.1038/s41419-019-1832-6)
Supplement: Supplementary file 1 — Supplemental materials and figures [file 41419_2019_1832_MOESM1_ESM.docx]

**Supplementary Materials and Methods**

**Studies by using human samples**

The primary colorectal cancer tissues and their matching, adjacent normal colon tissues were collected from 17 colorectal cancer patients undergoing surgery at the First Affiliated Hospital of Nanjing Medical University, Nanjing, China. Informed consent was given in all patients examined. All samples were confirmed by pathological examination. Histological grade was defined according to the World Health Organization classification. The First Affiliated Hospital with Nanjing Medical University Ethics Committee approved all described studies.

**Immunofluorescence (IF) staining**

The samples were permeabilized in 0.1%Triton X-100 and incubated with 1% BSA/PBS to block nonspecific binding. Subsequently, the cells were immunostained by incubating with HIBCH antibody (Abcam, ab153826; diluted 1:100), COX4 antibody (Santa Cruz Biotechnology, sc-376731; diluted 1:100) and p62 (diluted 1:100) overnight at 4°C. After being washed with PBS, cells were incubated with goat anti-rabbit AF594 (Thermo Fisher Scientific, Waltham, Massachusetts, A-11037; diluted 1:500); donkey anti-mouse AF488 (Thermo Fisher Scientific, R37114; diluted 1:500). And nuclei were counterstained with DAPI (Beyotime, Shanghai, China; C1006). Fluorescent images were taken and analyzed using the ZEN pro 2012 imaging software on a Zeiss invert microscope under 100- 630 fold magnification.

**TUNEL Assays**

TUNEL assays were performed according to the manufacturer’s instructions (Vazyme, A112, Jiangsu, China). In brief, tissue sections were prepared by fixing the tissue in 4% paraformaldehyde for 30 min at room temperature. A positive control sample was prepared by treatment with DNase I for 10 min at room temperature. The sections were pretreated with proteinase K for 10 min at room temperature and were washed with PBS. The slices were treated with Equilibration Buffer for 30 min at room temperature and were subjected to reaction with terminal deoxynucleotidyl transferase (TdT) enzyme and Alexa Fluor 488-conjugated dUTP at 37°C for 1 h. Nuclei were stained with DAPI for 10 min. Fluorescent images were taken and analyzed using the ZEN pro 2012 imaging software on a Zeiss invert microscope. The results were analyzed with ImageJ software.

**Apoptosis assay**

Cells were seeded into 6-well plates and exposed to DMSO or 1000 nM SBF-1 for indicated time. Apoptosis detected by Annexin V, FITC Apoptosis Detection Kit (DOJINDO, AD10, Kumamoto, Japan) according to the manufacturer’s instructions and analysis on an FACScalibur flow cytometer. For each cell line, single-stained and unstained controls were included.

**Western blot analysis**

Whole-cell lysates was prepared with Cell lysis buffer (Beyotime, P0013, Shanghai, China) containing protease and phosphatase inhibitors. Mitochondria isolation extracts steps were in accordance to the protocols provided by the manufacturer of Mitochondria Isolation Kit for Cultured Cells (Thermo Fisher, 89874, Massachusetts, USA). Equal amounts of cell lysates (25 μg) were loaded on SDS-PAGE and transferred onto PVDF membranes. After membranes were blocked, they were incubated with antibody against HIBCH(Abcam, ab153826; diluted 1:1000), LC3B (Cell Signaling Technology, 3868, Massachusetts, USA; diluted 1:1000), p-mTOR (Cell Signaling Technology, 2974; diluted 1:1000), COX-4 (Santa Cruz, sc-376731; diluted 1:1000), p-ULK1 (Ser757, Cell Signaling Technology, 14202; diluted 1:1000), p-ULK1 (Ser555, Cell Signaling Technology, 5869; diluted 1:1000), p-AMPK (Cell Signaling Technology, 2535; diluted; 1:1000), Tubulin (Proteintech, 10094-1-AP, Hubei, China; diluted 1:2000), p62 (Abcam, ab109012; diluted 1:1000) and Actin (Santa Cruz, sc-47778; diluted 1:4000), followed by incubation with goat anti-mouse IgG, goat anti-rabbit IgG HRP- conjugated antibodies were purchased from Santa Cruz Biotechnology. After extensive washing, the blots were developed with a chemiluminescence assay system (Cell Signaling Technology, 7003) and exposed to films (Kodak, NY, USA) for appropriate time periods.

**RNA isolation and quantitative real-time PCR**

Total RNA was extracted from the skin and cells using the TRIzol reagent (Invitrogen). First-strand cDNA was synthesized with 500 ng total RNA using a Hiscript® II QRTSuperMix (Vazyme, R122-01). Q-PCR was performed using iQ™ RT SYBR® Green supermix and the iQ5 real-time detection system (Bio-Rad Laboratories, 1708880, California, USA). The comparative cycle threshold (Ct) method was applied to quantify the expression levels through calculating the 2^(−ΔΔCt)^ method. The primers used for PCR were as follows: ACTB: 5′-ATTGGCAATGAGCGGTTC-3′ (forward) and 5′-GGATGCCACAGGACTCCAT-3′ (reverse); HIBCH: 5′-GCAATTTCGAGTGGCTACAGA-3′ (forward) and 5′- CCTTGGAGTCGTGGCAAGAA-3′ (reverse).

**Measurement of NAD^+^/NADH**

Cells were seeded into 6-well plates and exposed to DMSO or 1000 nM SBF-1 as well as transfected with scramble or shHIBCH for indicated time. The following steps according to the manufacturer’s instructions (Beyotime, S0175).

**Microscale Thermophoresis**

The interaction between SBF-1 and GFP-HIBCH (human HIBCH with an C-terminal GFP) was investigated by MST using a NanoTemper Monolith NT.115 instrument and the MO.AffinityAnalysis software (NanoTemper Technologies, Munich, Germany).

**Supplemental Figures**

**
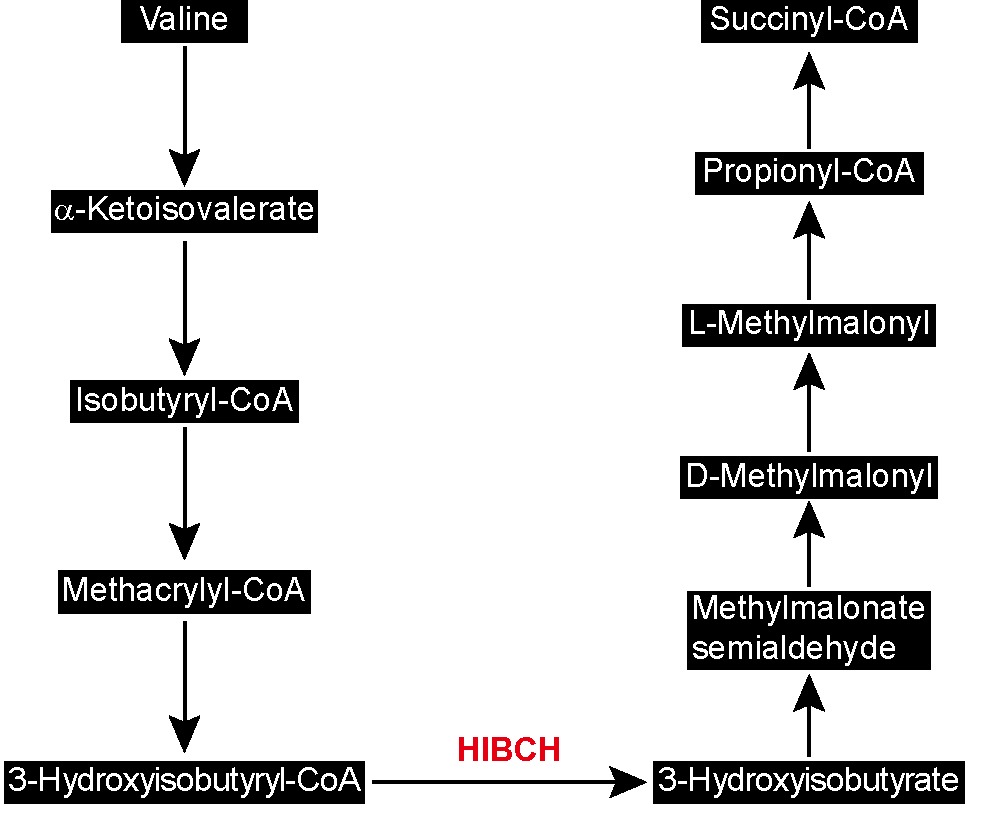
**

**Fig. S1** The schematic of valine catabolism pathway.


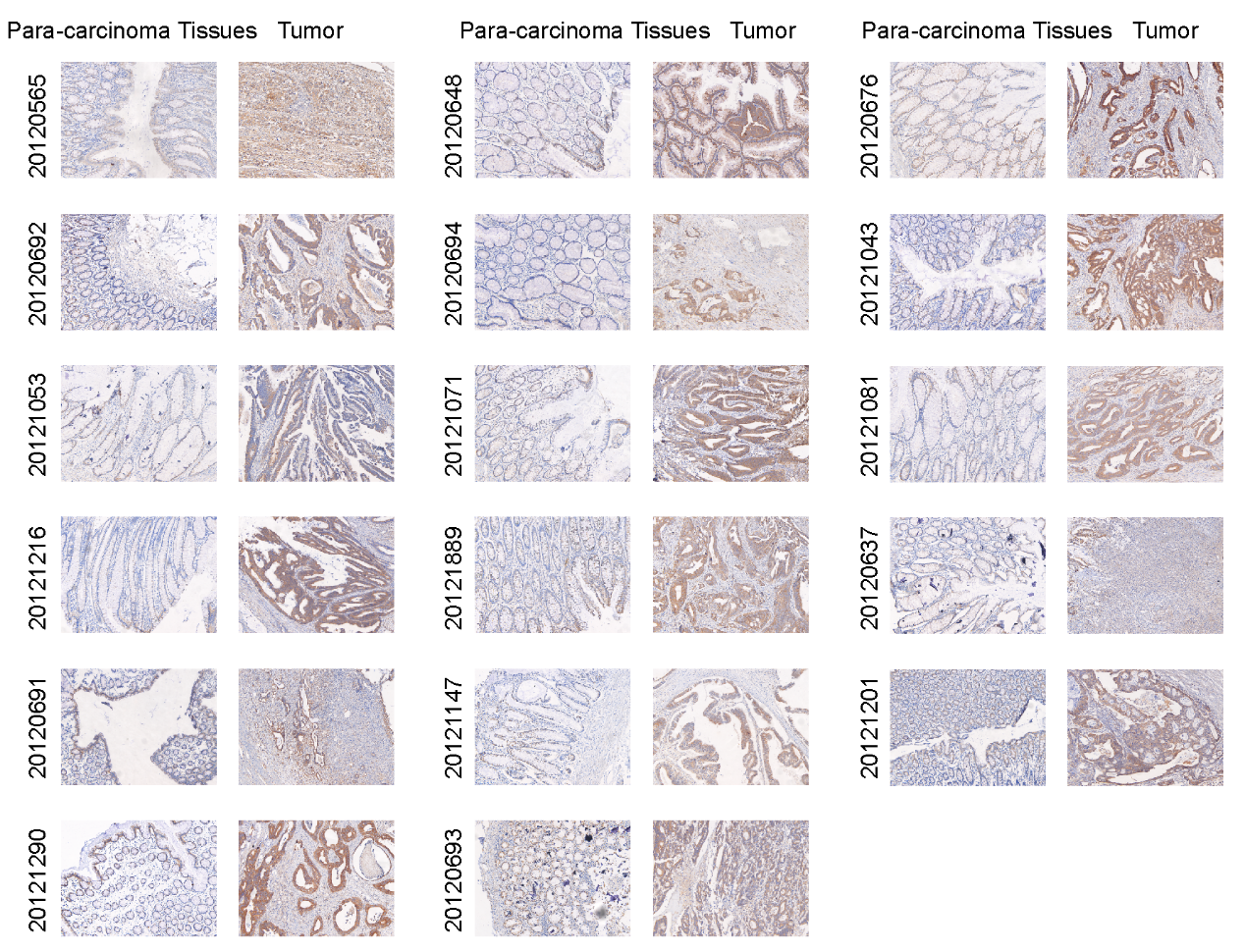


**Fig. S2** Immunohistochemical staining of para-carcinoma tissues and tumors from patients.


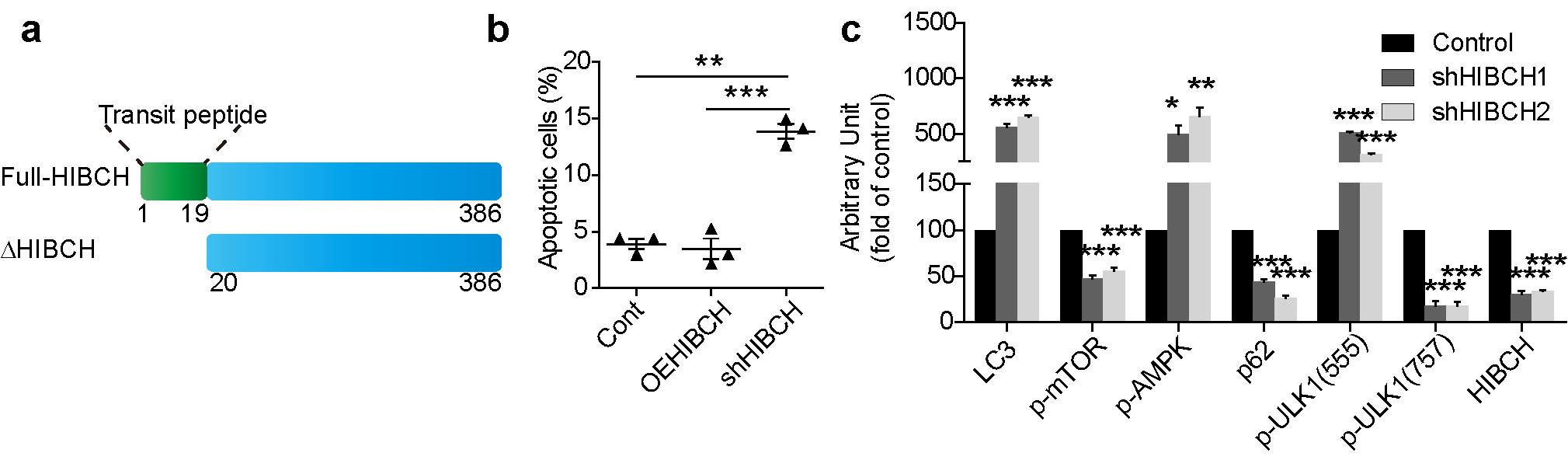


**Fig. S3** HIBCH is involved in CRC cell growth. (a) Schematic representation of the position and sequence of the transit peptide and the deletion of the transit peptide used for study. (b) HCT116 cells were transfected with various plasmids as described. Then the apoptosis was determined by Annexin V/PI staining. Percentage of Annexin V positive and PI positive cells was shown. *P* values were determined by one-way ANOVA with Tukey’s correction. (c) Relative band intensity was analyzed by Image J software. *P* values were determined by one-way ANOVA with Tukey’s correction. Data are mean ± SEM of three independent experiments. ******P*<0.05, *******P*<0.01, ********P*<0.001.


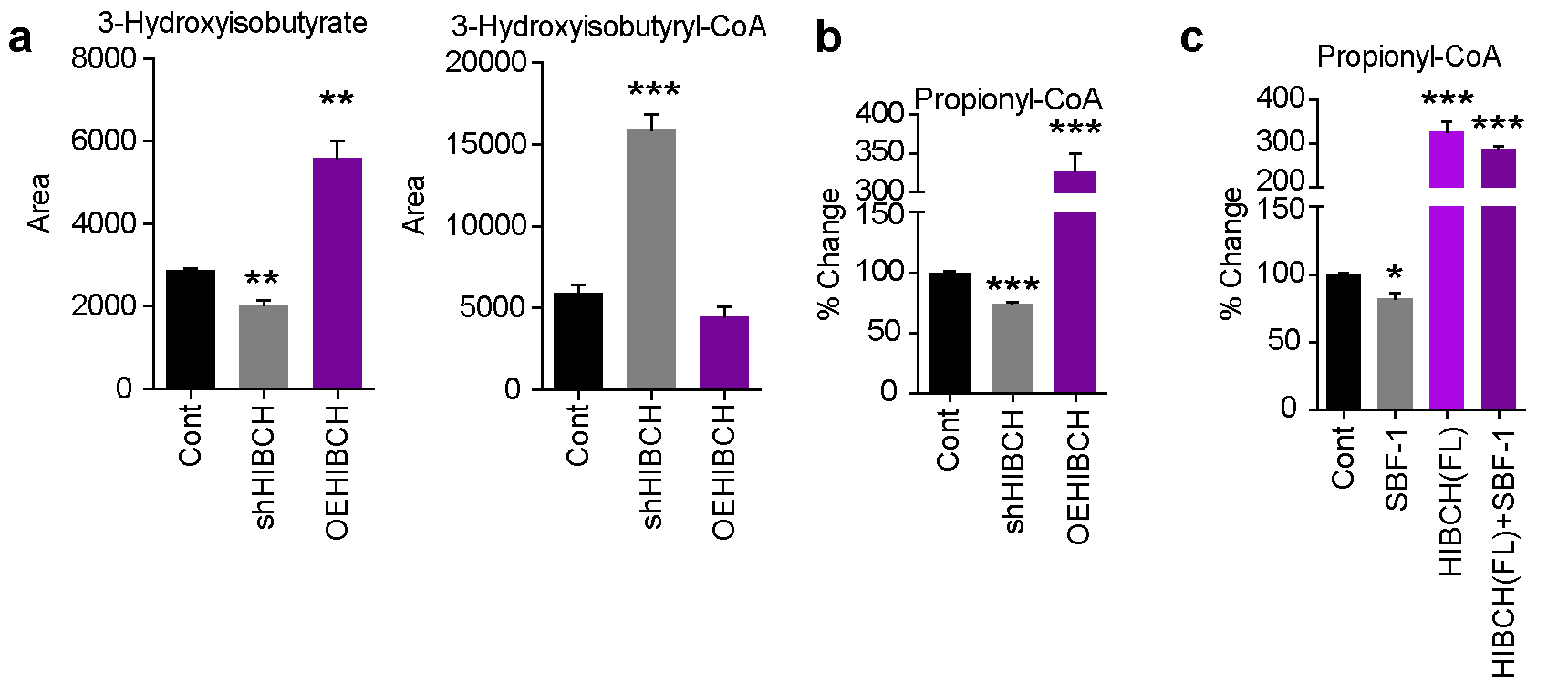


**Fig. S4** HIBCH deficiency results in decreased TCA cycle in CRCs. (a-b) HCT116 cells were overexpressed with HIBCH vector or interfered with HIBCH-shRNA for 48 h. (a) 3-Hydroxyisobutyryl-CoA (left) and 3-Hydroxyisobutyrate (right) levels in cells were measured by ultrafast UFLC-MS. *P* values were determined by Student’s t-test. (b) Propionyl-CoA levels were measured by UFLC-MS. *P* values were determined by Student’s t-test. (c) HCT116 cells were overexpressed with HIBCH vector for 24 h. Then cells were incubated with DMSO or SBF-1 (1000 nM) for 24 h. Propionyl-CoA levels in cell were measured by UFLC-MS as indicated treatment. *P* values were determined by Student’s t-test. Data are mean ± SEM of three independent experiments. ******P*<0.05, *******P*<0.01, ********P*<0.001.


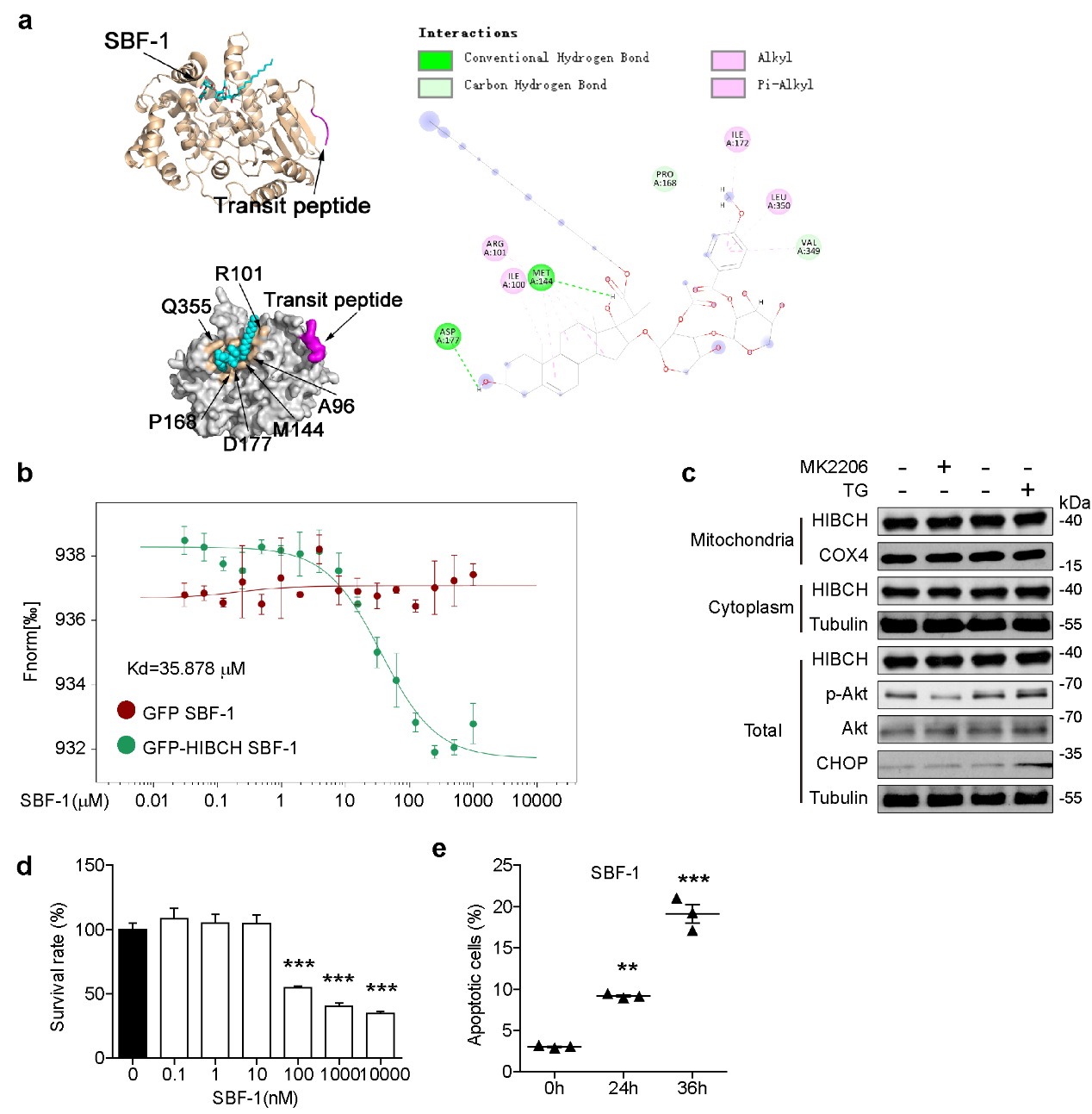


**Fig. S5** SBF-1 inhibits CRC cell growth. (a) A model of HIBCH with SBF-1 docked into the binding site. (b) The interaction of SBF-1 with GFP-tagged HIBCH was determined using MST. Data from three separate measurements were normalized and plotted, and a sigmoidal dose-response curve was fitted. Data are mean values ± SEM. (c) Cells were treated with of MK2206 (40 nM) or TG (5 μM) for 24 h. The mitochondria and the cytoplasm protein extract were subjected to immunoblot analysis for the detection of HIBCH localization. (d) HCT116 cells were seeded in 96-well plates and incubated with various concentrations of SBF-1 for 72 h. The inhibitory rate of SBF-1 on the cell proliferation was determined by MTT assay. Data are mean ± SEM of three independent experiments. *P* values were determined by one-way ANOVA with Tukey’s correction. (e) HCT116 cells were incubated with SBF-1 (1000 nM) for the indicated time. Then the apoptosis was determined by Annexin V/PI staining. Percentage of Annexin V positive and PI positive cells was shown. *P* values were determined by one-way ANOVA with Tukey’s correction. Data are mean ± SEM of three independent experiments. ***P*<0.01, ****P*<0.001.


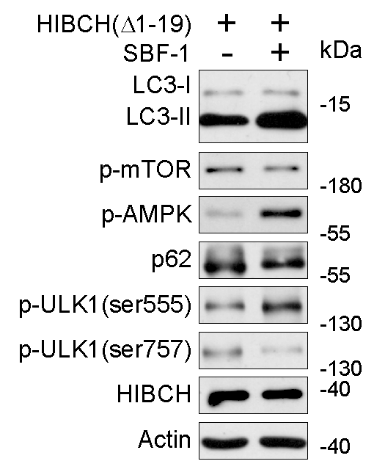


**Fig. S6** SBF-1 induces autophagy dependent on HIBCH. HCT116 cells were overexpressed with HIBCH for 24 h. Then cells were incubated with SBF-1 (1000 nM) for 24 h. The protein levels were determined by Western blotting.


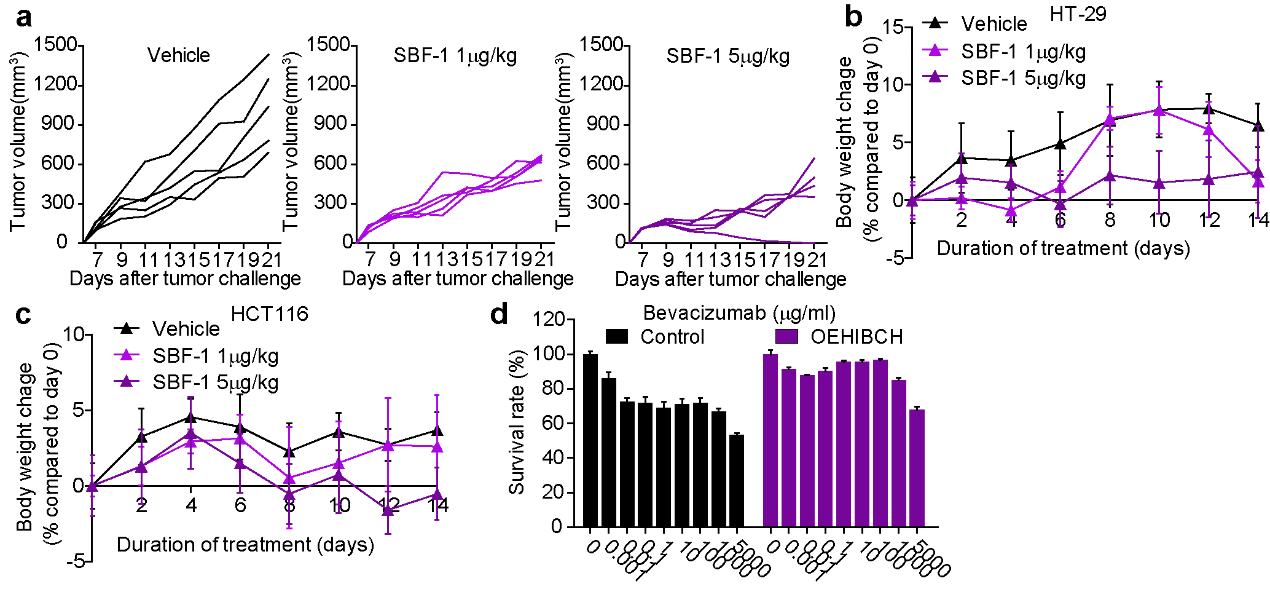


**Fig. S7** SBF-1 strongly inhibits the growth of human CRC xenografts in mice. (a) Growth of individual tumors from HT-29 xenograft tumors. (b) Relative body weights of mice bearing HT-29 subcutaneous xenograft tumors. n=5. (c) Relative body weights of mice bearing HCT116 subcutaneous xenograft tumors. n=5. (d) HCT116 cells were seeded in 96-well plates after transfection and incubated with various concentrations of Bevacizumab for 72 h. The inhibitory rate of Bevacizumab on the cell proliferation was determined by MTT assay. n=3. Error bars represent ± SEM.
